# Diagnostic mystery—a rare right ventricular cardiac hemangioma: a case report

**DOI:** 10.1186/s13019-021-01731-4

**Published:** 2021-12-31

**Authors:** Jingya Fan, Lei Guo, Peng Teng, Xiaoyi Dai, Qi Zheng, Shengjun Wu, Yiming Ni

**Affiliations:** Department of Cardiovascular Surgery, The First Affiliated Hospital, College of Medicine, Zhejiang University, 79# Qingchun Road, Hangzhou, 310003 Zhejiang China

**Keywords:** Cardiac tumor, Hemangioma, Right ventricle, Case report

## Abstract

**Background:**

Cardiac hemangiomas are rare in all kinds of benign cardiac tumors. Although cardiac hemangiomas affect all ages and may occur anywhere within the heart, right ventricular hemangiomas are extremely uncommon.

**Case presentation:**

We report a 56-year-old woman presented with chest tightness and breath shortness for 3 months. Transthoracic echocardiography and coronary computed tomography angiography showed a mass located adjacent to the apex of the right ventricle but both failed to figure out where the mass originated from, remaining a diagnostic mystery preoperatively. The mass was removed successfully and the histopathological examination confirmed it was hemangioma.

**Conclusions:**

Cardiac magnetic resonance should be the ultimate diagnostic tool of cardiac tumors. Surgical removal, associated with a low recurrence rate and long-term survival benefits, should be the first choice of therapy for cardiac hemangiomas.

## Background

Cardiac hemangioma, a type of benign heart tumor with a prevalence of < 2% among all cardiac tumors, is exceptionally rare [1]. Although cardiac hemangiomas affect all ages and may occur anywhere in the heart, right ventricular hemangiomas are extremely uncommon [2, 3]. Here we report one of these rare cases and discuss the diagnosis of heart tumors in the literature.

## Case presentation

**Fig. 1:**
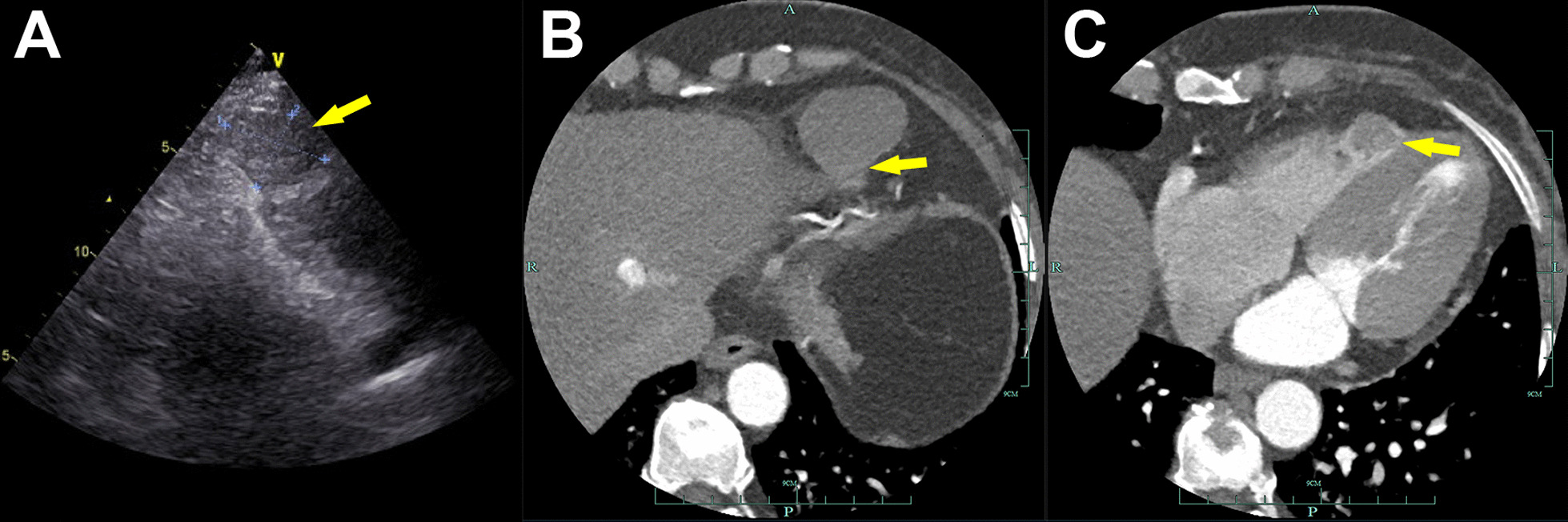
Preoperative imaging. **a** Re-transthoracic echocardiography showed the size of the mass as 4.0 × 3.0 cm(arrow); **b** Coronary CTA showed the mass has a clear boundary with the RV(arrow), but **c** showed an infiltrating mass appearing to be connected to the RV simultaneously (arrow)

A 56-year-old woman presented with a 3-month history of chest tightness and shortness of breath. Her symptoms gradually worsened, and she went to the local hospital, where transthoracic echocardiography showed an elliptically hypoechoic mass measuring approximately 4.0 × 2.8 cm located adjacent to the apex of the right ventricle (RV) in the pericardial cavity. It seemed to originate from the pericardium and had an unclear border with a normal right ventricular myocardium; no obvious blood flow signal was detected in the mass. Re-transthoracic echocardiography in our institution identified the size of the mass as 4.0 × 3.0 cm (Fig. 1A), which was similar to the result from the local hospital. Coronary computed tomography angiography (CTA) was performed for further diagnosis, but it did not identify the origin of the mass. The tumor showed a clear boundary with the RV (Fig. 1B), suggesting its pericardial origin, while the tumor appeared to be connected to the RV on another image (Fig. 1C). It was diagnosed preoperatively as a tumor originating from the pericardium and invading the RV through discussion, but the possibility of malignancy could not be completely excluded. Consequently, a median sternotomy surgery was performed under cardiopulmonary bypass.

**Fig. 2:**
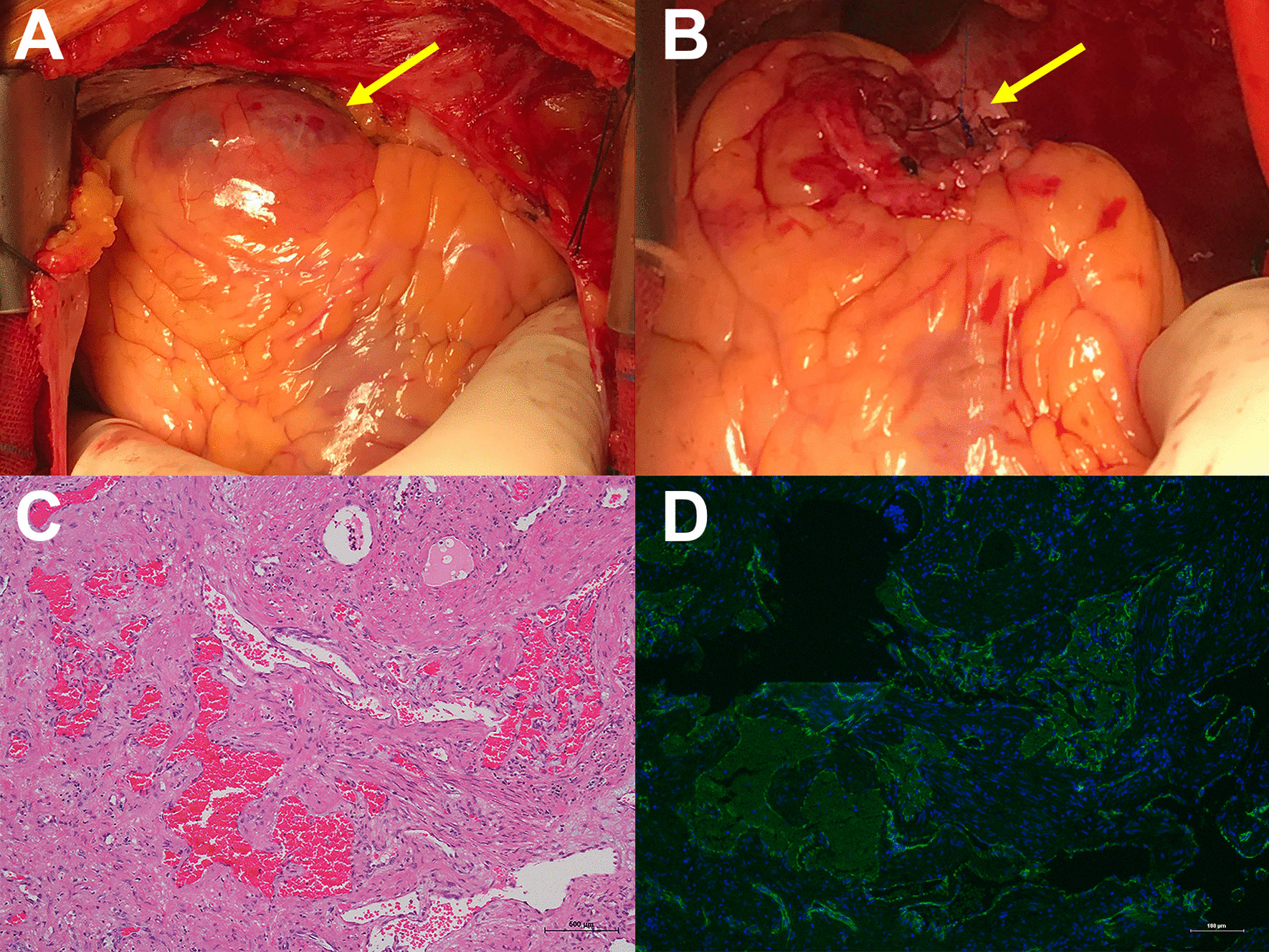
Intraoperative macroscopic findings and histopathological examination. **a** Intraoperative macroscopic findings showed a smooth and bulky tumor measuring 4 cm × 3 cm × 3 cm originated from the apex of the RV and adhered to the pericardium (arrow); **b** The tumor was completely removed and the defect was sutured and enforced with a patch of autologous pericardium along the incision line (arrow); **c** Histopathological examination (Hematoxylin–eosin, × 40) and **d** Immunohistochemical staining for CD31 confirming it was hemangioma

After incising the pericardium, we found that the tumor (4 × 3 × 3 cm) originated from the apex of the RV and adhered to the pericardium (Fig. 2A). A part of the capsule protruded out of the heart, while the rest was integrated and communicated with the RV myocardium. The normal ventricular myocardium was thin at the boundary between the two. A solid mass in the tumor, which was filled with bloody cyst fluid was observed, but myocardial ischemia was not observed. The tumor was completely removed, and the defect in the RV was sutured and enforced with a patch of autologous pericardium along the incision line (Fig. 2B). The postoperative pathological diagnosis was hemangioma (Figs. 2C, D). The patient had an uneventful recovery without complications and was discharged on the seventh postoperative day. No signs of recurrence or right heart dysfunction was observed on thoracic echocardiography during 4 months of postoperative follow-up.

## Discussion and conclusion

Cardiac hemangiomas represent only 1–2% of all benign heart tumors [1]. Most affected patients are asymptomatic, but symptoms that do occur depend on the tumor’s location and size and are always non-specific, such as dyspnea, arrhythmia, angina, signs of right heart failure, and thromboembolic events [4, 5]. Consequently, cardiac hemangiomas are often discovered by transthoracic echocardiography and misdiagnosed as other cardiac neoplasms (e.g., cardiac myxoma) [6]. The right ventricular hemangioma is extremely rare, especially at the apex of the RV. According to Jiang et al. [3], the most common site of right ventricular hemangiomas is the anterior wall of the RV, but only 6.7% are located at the apex of the RV. In our case, the hemangioma was located at the apex of the RV and grew outward reaching 4.0 cm, which was different from most cardiac hemangiomas, which were single, relatively small subendocardial nodules (2.0–3.5 cm) [7]. These characteristics significantly increased the difficulty of diagnosis.

Diagnostic tools for cardiac tumors mainly include echocardiography, chest computed tomography (CT), and cardiac magnetic resonance (CMR) imaging. Transthoracic echocardiography is the preferred diagnostic tool for cardiac tumors because of its non-invasiveness and convenience; however, it cannot accurately distinguish the tissue level and it cannot display the blood supply to the tumor unless contrast-enhanced ultrasound is applied. Considering that there was no obvious blood flow signal in the mass on echocardiography, we deemed it to be of non-cardiac origin preoperatively. Contrast-enhanced CT may compensate for these shortcomings, but it is unfavorable to patients who are allergic to contrast agents or with renal insufficiency.

Coronary CTA and coronary angiography are also used to show the distribution of vessels, feeding vessels to the tumor, and whether the coronary arteries are oppressed [8]. In our case, the origin of the tumor remained a mystery in the result of two coronary CTA images associated with key information of the origin, revealing quite different findings, leaving us in a diagnostic dilemma. If we used CMR at that time, we might determine the properties of the tumor and its relationship with the RV anterior free wall and pericardium. The excellent contrast resolution and multiplanar capability of CMR imaging allows for qualitative diagnosis and optimal anatomical evaluation of any cardiac tumor. In addition, CMR imaging enabled us to demonstrate the precise relationship among the tumor, tricuspid valve, and RV anterior free wall, which is useful for pre-surgical planning [5]. However, the implantation of pacemakers or metal objects, such as biliary stents, and the high price limit the application of CMR in our country.

Surgical removal is the first choice of treatment for cardiac hemangiomas [9]. After complete resection, the prognosis is generally favorable, with a low recurrence rate. Furthermore, an incomplete resection has been reported to produce long-term survival benefits [4].

In our case, since the hemangioma at the apex of the RV is extremely rare, and its diagnosis is difficult, we could not determine the properties of the tumor using echocardiography and CT investigations. CMR should be the ultimate method when diagnosing cardiac tumors that are difficult to be determined. The patient in this case was successfully operated, and there was no recurrence or other complications in the subsequent follow-up.

## Data Availability

Please contact author for data requests.

## Abbreviations

RV: Right ventricle
CTA: Computed tomography angiography
CT: Computed tomography
CMR: Cardiac magnetic resonance
